# Identification of promising SARS-CoV-2 main protease inhibitor through molecular docking, dynamics simulation, and ADMET analysis

**DOI:** 10.1038/s41598-025-86016-9

**Published:** 2025-01-22

**Authors:** Ganesh Sharma, Neeraj Kumar, Chandra Shekhar Sharma, Taha Alqahtani, Yewulsew Kebede Tiruneh, Sharifa Sultana, Gabriel Vinícius Rolim Silva, Gabriela de Lima Menezes, Magdi E. A. Zaki, Jonas Ivan Nobre Oliveira

**Affiliations:** 1 Department of Pharmaceutical Chemistry, Bhupal Nobles’ College of Pharmacy, Bhupal Nobles’ University, Udaipur, 313002 India; 2 Department of Pharmacology, College of Pharmacy, King Khalid University, Abha, 62529 Saudi Arabia; 3 Department: Biology, Biomedical Sciences stream Bahir Dar University, Bahir Dar, P.O.Box=79, Bahir Dar, Ethiopia; 4 Department of Biophysics and Pharmacology, Bioscience Center, Federal University of Rio Grande do Norte, Natal, 59064-741 RN Brazil; 5 Department of Chemistry, College of Science, Imam Mohammad Ibn Saud Islamic University (IMSIU), Riyadh, Saudi Arabia

**Keywords:** COVID-19, Mpro, Molecular Docking, SARS-CoV-2, 3CLpro, Molecular dynamics, ADMET, Biophysics, Computational biology and bioinformatics

## Abstract

The COVID-19 pandemic caused by SARS-CoV-2 continues to pose a major challenge to global health. Targeting the main protease of the virus (Mpro), which is essential for viral replication and transcription, offers a promising approach for therapeutic intervention. In this study, advanced computational techniques such as molecular docking and molecular dynamics simulations were used to screen a series of antiviral compounds for their potential inhibitory effect on the SARS-CoV-2 Mpro. A comprehensive analysis of compounds from the ChemDiv and PubChem databases was performed. The physicochemical properties, pharmacokinetics, and ADMET (Absorption, Distribution, Metabolism, Excretion, and Toxicity) profiles were evaluated to determine drug similarity and safety. Compound 4896 − 4038 proved to be the most promising candidate. It exhibited a favorable balance between molecular weight (491.06) and lipophilicity (logP 3.957), high intestinal absorption (92.119%), and broad tissue distribution (VDss of 0.529), indicating good oral bioavailability and therapeutic potential. Molecular docking studies showed that 4896 − 4038 has a strong binding affinity to the active site of Mpro and forms key interactions, such as hydrogen bonds, carbon-hydrogen bonds, pi-sulfur, and multiple van der Waals and pi-pi stacked bonds. The binding energy was comparable to that of the reference drug X77, indicating potential efficacy. Molecular dynamics simulations over 300 ns confirmed the stability of the Mpro/4896 − 4038 complex of protein-ligand. Free energy landscape mapping and MM/PBSA calculations further substantiated the favorable binding and stability of the complex. Importantly, 4896 − 4038 exhibited a comparatively favorable safety profile. In summary, compound 4896 − 4038 shows significant potential as a potent SARS-CoV-2 Mpro inhibitor, combining potent inhibitory activity with favorable pharmacokinetic and safety profiles. These results support the further development of 4896 − 4038 as a promising therapeutic agent in the fight against COVID-19 that warrants experimental validation and clinical investigation.

## Introduction

Coronavirus disease 2019 (COVID-19) is a highly contagious respiratory illness caused by the novel coronavirus SARS-CoV-2^1^. The conclusion of 2019 and the commencement of 2022 have been profoundly disrupted by a pandemic that has since been identified as COVID-19, and its global impact persists to the present day. The statistics provided by the World Health Organization (WHO)^2^indicate that there have been more than 403 million cases and over 6.3 million fatalities. The initial case began in the Central region of China, namely in Wuhan, and has since been disseminated globally^3^. The globe is currently facing a pandemic scenario that has caused concern and prompted the implementation of preventative measures and diagnostics, as recommended by the WHO. “Prevention is superior to treatment,” and today, prevention is the only viable option until a definitive cure is found^4^. The implementation of rigorous cleanliness protocols, strict social distancing measures, and widespread lockdowns has resulted in a near-complete halt to global functioning, leading to significant economic repercussions^5^. The availability of anti-viral medications is limited, and historically, vaccinations have been relied upon more heavily to protect humanity against such disease-causing agents^6^. The current situation has become more alarming due to the unique form of the virus and the absence of any known prior therapy^7^. In these critical circumstances, the scientific community has been compelled to investigate previously documented medications through the process of “repurposing”^8^. To focus on a specific method, preference has been given to natural products (e.g., plants, organisms, marine sources, etc.) instead of artificial substances. Researchers are now investigating the potential of natural alkaloid noscapine and other related antiviral medications for the treatment of COVID-19.

Coronaviruses are a specific type of virus that has a single-stranded positive-sense RNA ((+) ssRNA) structure. The coronaviruses are classified into four different groups: alpha, beta, delta, and gamma coronaviruses^9^. The structural information of the virus consists of spike glycoproteins (S protein), nucleocapsid protein (N protein), matrix proteins (M protein), and envelope proteins (E protein)^10,11^. According to several studies and genomic studies, it has been suggested that the main protease of SARS-CoV-2 plays a vital role in viral replication, transcription, and regulation. Therefore, it is regarded as a promising target for therapeutic design^9^.

The lung is the target site where the coronavirus infects the most^12^. However, due to the widespread presence of ACE2 receptors in other organs, such as the cardiovascular system, liver, kidneys, gastrointestinal tract, eyes, and central nervous system, it is important to regularly monitor for potential damage in these areas^2,13^. The antiviral medicine has been extensively evaluated over the past two decades and has been found to have minimal toxicity and no effect on natural humoral or adaptive immunity^14^. Furthermore, it has been proven to have minimal toxicity against organs and tissues. Collectively, these results emphasized the significance of antiviral drugs and their antiviral capabilities, which prompted interest in assessing their effectiveness against SARS-CoV-2^15^.

In light of the current pandemic and the pressing need for effective antiviral drugs, we have been using advanced molecular modeling techniques to investigate potential compounds that have exceptional selectivity against COVID-19. In addition, we have conducted a comparison between the antivirals in our library and the previously identified ligand (X77) that has shown promising therapeutic effects against COVID-19. This comparison aims to enhance the effectiveness of repurposing the antiviral library. We utilized sophisticated computational techniques, including molecular docking and molecular dynamics (MD) simulations. Molecular docking investigations have been widely employed to study and understand the molecular interaction between medicines and specific protein receptors^16^. On the other hand, MD studies provide insights into the atomic fluctuations of complex interaction systems through parameters such as RMSD, RMSF, and others^17^.

Computer-aided drug design (CADD) plays a vital role in contemporary drug research and development^18^. To strike a balance between computational efficiency and accuracy, a hierarchical method is employed, integrating several scoring systems in both the drug lead discovery and optimization phases. The recently released crystal structure of the SARS-CoV-2 main protease provides a dependable basis for the identification of drugs that might potentially interact with this particular protein target^19^. In this investigation, they employed multiscale modeling techniques to identify possible agents that may be repurposed to selectively target the major protease of SARS-CoV-2.

Several research institutions are expediting the process of discovering vaccines and therapeutics for COVID-19, highlighting the urgency of finding new treatment options for this disease. This study employed pharmacokinetics and molecular docking analysis to investigate the interaction between eleven ligands and a SARS-CoV-2 protease using computational methods, as well as their pharmacological efficiency and toxicity profiles. The crystallographic structure of SARS-CoV-2 major protease (Mpro) was used.

While there is currently other work using molecular docking and molecular dynamics methods to investigate the efficacy of antiviral therapy options for COVID-19, few have looked at the ADMET profiles of the individual compounds, and even fewer at their quantum mechanical properties. In our work, we, therefore, aimed not only to perform a comprehensive analysis of the binding properties of each potential new antiviral and the already well-studied X77 but also to investigate their quantum energetic profiles and the impact of these energies on their efficacy. Our comprehensive analysis aimed to investigate the interactions between each ligand and the Mpro protease, to identify potential therapeutic candidates for COVID-19.

## Material and method

### Hardware and Software

The computational study was performed on Windows 10 (64-bit) operating systems equipped with 8 GB of RAM and a 2.11 GHz Intel^®^ Core™ i5-10210U processor, except for the MD simulations. For the MD simulations, a Supermicro AMD with NVIDIA GPU and 80 GB of RAM was used to run the GROMACS 2024 software.

## SARS-CoV-2 main protease (Mpro) retrieval and evaluation

Although the association between Mpro/X77 and the antiviral library of ligands is well established, the structures for Mpro and X77 were obtained from the protein data bank (PBD) (PDB ID: 6W63) and used in docking studies as a target Mpro. These structures were checked for abnormalities using the Autodock tool and then prepared for docking simulations with antivirals. For the docking simulation, the receptor was prepared by including the solvation parameters, Kollman united atom charges, and polar hydrogen in the protein. As molecules are not peptides, the Gasteiger charge was ascribed to them, and then non-polar hydrogens were combined. Autodock incorporates pre-computed grid maps, one for each kind of atom found in the ligand being docked, which holds the corresponding potential energy. The macromolecule’s active site must be included in the grid, as stated by Morris and his team^20^. The protein-ligand combination underwent geometric optimization using the UFF force field, as described by Jorgensen and Tirado in 2005.

## Drug-likeness, pharmacokinetics, and ADMET properties analysis

An extensive analysis was conducted employing computational platforms to evaluate the drug-likeness, pharmacokinetics, and ADMET (Absorption, Distribution, Metabolism, Excretion, and Toxicity) features of all compounds, as well as the antiviral X77. For this purpose, the canonical SMILES and sdf of each drug was obtained from data banks such as ChemDiv (https://www.chemdiv.com/*)* and PubChem (https://pubchem.ncbi.nlm.nih.gov/*).* The webservers ADMETlab 3.0 (https://admetlab3.scbdd.com/), FAF-Drugs 4.1 (https://mobyle2.rpbs.univ-paris-diderot.fr/cgi-bin/portal.py#forms::FAF-Drugs4), Deep-PK (https://biosig.lab.uq.edu.au/deeppk/*)*, pkCSM (https://biosig.lab.uq.edu.au/pkcsm/prediction), vNN-ADMET (https://vnnadmet.bhsai.org/vnnadmet/home.xhtml), Pred-hERG 5.0 (http://predherg.labmol.com.br/), and ADVERPred (https://www.way2drug.com/adverpred/*)* were used to obtain a range of drug-likeness and ADMET-related properties for each compound.

## Ligand geometry optimization

All compounds were first extracted from the ChemDiv (https://www.chemdiv.com/) and PubChem (https://pubchem.ncbi.nlm.nih.gov/) libraries and then subjected to geometric optimization to prepare them for the docking procedures using Avogrado (http://avogadro.cc), ChimeraX (https://www.cgl.ucsf.edu/chimerax/) and SwissDrugDesign (https://www.molecular-modelling.ch/swiss-drug-design.html).

The geometry of each ligand was optimized using the CHARMM27 force field. A conformational analysis was performed to generate up to 500 conformers per molecule with an RMSD cutoff of 0.5 Å. The most stable conformers were selected for subsequent quantum chemical calculations by Gaussian 16 Rev. C.01 software (https://gaussian.com/).

The density functional theory (DFT) optimizations and calculations were performed using the basis set B3LYP/6-31G(d, p), supported by simulations of solvent effects aiming to mimic biological conditions^21,22^. The main goal of this step is to obtain ligands that are geometrically optimized, which is crucial for the energetic and quantum chemical characterization (molecular docking and molecular dynamics simulations) of ligands (ligand-receptor complexes)^23–27^.

## Ligands preparation and molecular docking

Both ligands and proteins underwent preparation prior to the docking analysis. For the protein, water molecules were removed, and structural imperfections—such as missing side-chains or regions of structural disorder—were corrected using MolProbity, a comprehensive structural validation tool. During this step, we utilized MolProbity (http://molprobity.biochem.duke.edu/), a comprehensive structural validation tool, to identify and address potential issues in the protein structure. MolProbity facilitated the addition of missing hydrogens, optimization of hydrogen-bond networks, and evaluation of the protein geometry through Ramachandran plots and rotamer analyses. Furthermore, the tool enabled the identification and correction of flipped Asn, Gln, and His side-chains, ensuring the structural integrity and suitability of the receptor for docking. Subsequently, the protein’s backbone was fixed, and any missing hydrogen atoms were added. The system was further minimized using the CHARMm force field to eliminate residual strains and steric clashes. MolProbity’s steric clash analysis was instrumental in refining the structure by resolving all-atom contacts that could otherwise interfere with docking accuracy, ensuring a robust and reliable receptor model for computational analysis. Docking simulations were performed using SwissDock (https://www.swissdock.ch/), leveraging EADock DSS and Attracting Cavities 2.0 (AC) algorithms. The binding site was defined based on the X77 ligand-binding pocket observed in the co-crystallized Mpro complex, with a 6.0 Å sphere delineating the docking region to focus the analysis^28^. Docking poses were evaluated using two complementary scoring functions: (i) the AC docking score, integrating CHARMM force field energy and FACTS solvation energy terms, and (ii) the SwissParam score, estimating binding free energy as a weighted sum of polar and nonpolar contributions. While AC docking scores prioritized ligand ranking, SwissParam scores provided a comparative framework for multiple candidates, ensuring robust selection of the most promising compounds. After docking, binding poses and parameters were analyzed with PLIP (https://plip-tool.biotec.tu-dresden.de/plip-web/plip/index), LigPlot+ (https://www.ebi.ac.uk/thornton-srv/software/LigPlus/) and PoseView (https://proteins.Plus/), namely number, angle and distance of intermolecular hydrogen bonds (conventional, carbon and pi-donor H bonds), electrostatic (attractive charges, salt bridge, pi cation, pi anion), hydrophobic (pi-pi stacked, pi-pi t-shaped, amide-pi stacked, alkyl, pi-sigma, pi-alkyl), halogen (fluorine, Cl, Br and I), miscellaneous (steric bumps, charge repulsion, acceptor-acceptor clashes, metal repulsion) and unfavorable interactions.

### Molecular docking validation

MolProbity’s role in optimizing hydrogen networks and protonation states added further reliability to receptor-ligand binding predictions. The reliability of the docking results was validated through computational and structural assessments. SwissDock’s integration of Attracting Cavities 2.0 and AutoDock Vina, both extensively benchmarked algorithms, ensured precise ligand-receptor interaction predictions. The dual scoring functions further enhanced result accuracy by capturing interaction energies and binding affinities comprehensively. Validation efforts included verifying binding poses against known structural data. The binding site’s definition—based on the X77 ligand-binding pocket—provided experimentally validated spatial constraints, ensuring biologically relevant docking simulations. Predicted molecular interactions, such as hydrogen bonds, hydrophobic contacts, and π-stacking interactions, were consistent with established molecular recognition principles^29–35^.

## MD analysis of 4896 − 4038 and X77 with Mpro enzyme

The MD simulation was performed with the GROMACS 2024 software^36,37^in a High-Performance Cluster (HPC) environment. The Mpro enzyme was subjected to force field processing with AMBER99SB-ILDN^38^ all-atom. For ligand parameterization, we used the ACPYPE tool on the https://www.bio2byte.be web server. A minimum of 12 angstroms (Å) separated the protein atom from each box edge when the starting structure was placed in a cubic box. TIP3P water was used to dissolve this box and ions were used to neutralize the system.

The LINCS algorithm^39^was used to constrain the covalent bonds of the solutes with hydrogen atoms, while the SETTLE algorithm^40^was used to obtain the internal rigid structure of the solvent molecules (water). The V-rescale^41^and Parrinello-Rahman^42^ algorithms were used to regulate the temperature and pressure of the system, which were set to 310 K (36.85 °C) and 1 atm, respectively. For long-range electrostatic interactions, the Ewald summation method for particle networks was applied, and a limit of 1.0 nm was set for non-bonded interactions.

The system was first subjected to two energy minimization procedures. In the first procedure, the protein positions were restricted and 500 steps of the steepest descent algorithm were used. The identical algorithm, but with 10,000 steps and flexible water, was used in the second minimization step. After the minimization steps, the system underwent two 100 picoseconds (ps) phases of equilibration: an NVT ensemble and an NPT ensemble with protein position constraint to equilibrate the thermodynamic variables. Without protein limitation, the third and final equilibration phase was performed as an NPT ensemble with a duration of one nanosecond (ns).

Finally, an NPT ensemble performed the production run at 310 K for a total duration of 300 ns. The MD equations of motion with 2 fs as a time-step were integrated using the leap-frog^43^ approach. GROMACS 2024 was used for the trajectory analysis and the graphs were generated using the GROMACS tool commands gmx.

After all solvent molecules and ions were removed, the last 500 frames (50 ns) of the complexes were analyzed using Molecular Mechanics/Poisson-Boltzmann surface area (MM/PBSA) calculation. In addition, MM/PBSA decomposition analysis was performed per residue to evaluate the energy contribution of the residue. The software used for these analyses was the recently developed tool ‘gmx_MMPBSA’^44^, which allows the user to calculate the MM/PBSA directly from the output of GROMACS MD.

## Results

### Pharmacokinetic and ADMET analysis

The comprehensive evaluation of physicochemical and pharmacokinetic profiles is crucial for the rational design and development of new therapeutic agents, aiming to assess their efficacy^45–47^. This analysis provides valuable insight into the potential biological activity, efficacy and safety profiles of drug candidates by expanding on their pharmacokinetic properties. Here, we focused on the antiviral compounds 4896 − 4038, 4903 − 2142, E859-0698, C736-0680, 8012 − 1209, 6392 − 0766, D361-0772, C530-1227, E565-0117 and K306-0164 and the reference drug X77 and evaluated their suitability as SARS-CoV-2 Mpro inhibitors based on their physicochemical and pharmacokinetic attributes.

**Fig. 1:**
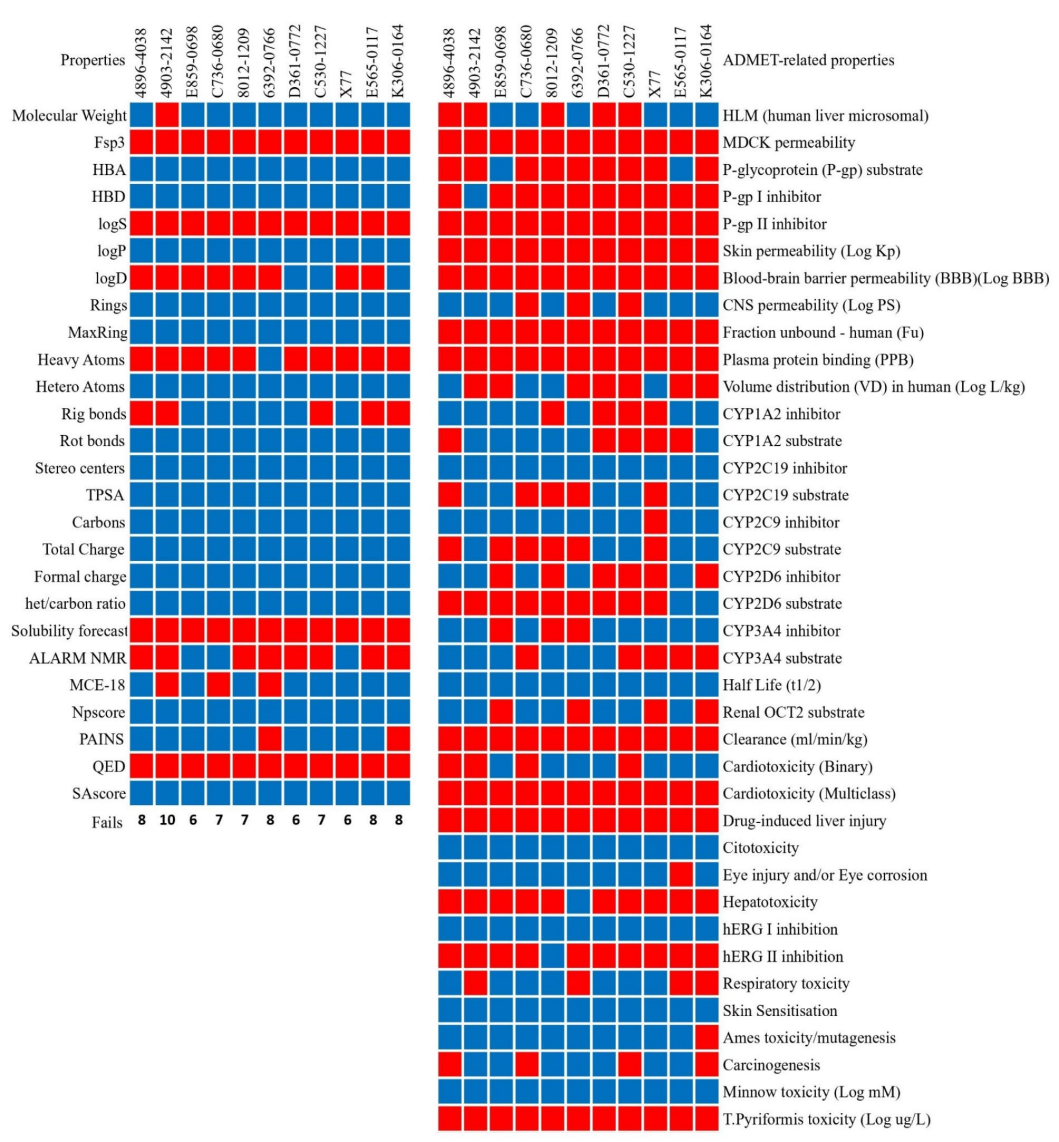
**-** Physicochemical (left) and ADMET (right) properties for each compound. Blue denotes values that fit within acceptable ranges, with red falling short of them. For the CYP enzymes family, blue denotes interaction and red denotes non-interaction as a substrate and/or inhibitor.

The molecular weight (MW) of the compounds varies, with 4896 − 4038 being 491.06 g/mol and others ranging from 383.15 g/mol (C736-0680) to 534.18 g/mol (4903 − 2142). The reference drug X77 has a MW of 459.26 g/mol. According to Lipinski’s Rule of Five, a molecular weight below 500 g/mol is favorable for oral bioavailability^48^. All compounds fulfill this criterion except 4903 − 2142, which with a molecular weight of 534.18 g/mol exceeds the limit, which could impair its absorption. A comparison between this property can be seen in Fig. 1.

The lipophilicity, indicated by the LogP values, varies between the compounds. Compounds C736-0680 (LogP 4.962), 4903 − 2142 (LogP 4.957), and E859-0698 (LogP 4.852) have higher lipophilicity, which could improve membrane permeability, but could have a negative effect on solubility and increase plasma protein binding^49^. Compound 6392 − 0766 has the highest LogP value of 5.27, which exceeds the recommended limit and may affect the drug property. On the other hand, compounds D361-0772 (LogP 2.855) and C530-1227 (LogP 2.958) are more hydrophilic, indicating better solubility and potentially improved absorption. The reference drug X77 has a LogP of 3.811, which is within the acceptable range (Fig. 1).

Hydrogen bond donors (HBD) and acceptors (HBA) play an important role in drug-receptor interactions. For most compounds, the HBD and HBA values are within the recommended limits of Lipinski’s Rule of Five (HBD ≤ 5, HBA ≤ 10). Compounds such as 8012 − 1209 (HBD 2, HBA 8) and D361-0772 (HBD 1, HBA 10) fulfill these criteria. X77, the control compound, has an HBD of 2 and an HBA of 7. Overall, all but one compound (Compound 4903 − 2142) fulfilled Lipinski’s Rule of Five, with 4903 − 2142 exceeding the recommended MW value of 500; however, since the rule allows for a maximum of one violation, we can imply that all compounds meet the limits, indicating a favorable drug-likeness profile^48^.

The topological polar surface area (tPSA) values for the compounds range from 55.11 Å² (C736-0680) to 113.84 Å² (8012 − 1209) and are all below the maximum recommended value of 140 Å² for optimal intestinal absorption, suggesting favorable bioavailability. The reference drug X77 has a tPSA value of 90.98 Å². The number of rotatable bonds (nRotb) varies between 3 (4896 − 4038 and K306-0164) and 7 (4903 − 2142), all of which are below the Veber’s Rule limit of 10, indicating potentially good oral bioavailability. According to Veber’s Rule, both tPSA and the number of rotatable bonds are critical for oral bioavailability, and all compounds fulfill these criteria (Fig. 1)^50^.

The fraction of sp³ carbons (Fsp³) varies between compounds, reflecting differences in three-dimensionality and saturation^51^. X77 has a higher Fsp³ value of 0.407, indicating a greater molecular complexity that may favor biological interactions. Among the compounds tested, 8012 − 1209 (Fsp³ 0.286), D361-0772 (Fsp³ 0.269) and C530-1227 (Fsp³ 0.231) have higher Fsp³ values, which could contribute to better solubility and bioavailability. Compounds with lower Fsp³ values, such as 4896 − 4038 (0.083) and 6392 − 0766 (0.077), may have less three-dimensional character, which could affect their pharmacokinetic properties^51^. However, all compounds fell short of complying with the minimum value for Fsp³ at 0.42 (Fig. 1).

The QED (Quantitative Estimate of Drug-likeness) values for the compounds range from 0.231 (4903 − 2142) to 0.558 (C530-1227), with higher values indicating better drug-likeness. Compounds C530-1227 and 8012 − 1209 have the highest QED values of 0.558 and 0.556, respectively, indicating promising drug-like properties^52^. The reference drug X77 has a QED value of 0.546. In general, a QED value closer to 1 indicates a higher probability of success in drug development, so although no compounds met the mean value of 0.67^52^ for attractive compounds, C530-1227 and 8012 − 1209 were the drugs with the highest QED value and also closest to our reference drug X77, showing these compounds have potential.

The ADMET analysis for each compound yielded a critical look into their pharmacokinetic behavior and potential efficacy. In terms of absorption, most compounds exhibit good permeability in the Caco-2 assay. Compounds C736-0680, X77, E859-0698, and K306-0164 have higher permeability values between 1.353 and 1.574, indicating efficient passive permeability through intestinal epithelial cells and better intestinal absorption. In contrast, compounds 4903 − 2142 and 8012 − 1209 have lower permeability values of 0.488 and 0.486, respectively. In terms of human intestinal absorption (HIA), all compounds exhibit high values above 90%, with D361-0772 and K306-0164 reaching 100%, indicating excellent potential for oral bioavailability. This comparison can be seen in Fig. 1.

P-glycoprotein (P-gp) interactions are critical for drug absorption and disposition^53^. Most compounds, including 4896 − 4038, 4903 − 2142, C736-0680, 8012 − 1209, 6392 − 0766, D361-0772, C530-1227, X77, and K306-0164, have been identified as P-gp substrates, which may lead to decreased absorption due to efflux mechanisms and contribute to multidrug resistance. In particular, compounds 4896 − 4038, C736-0680, 8012 − 1209, 6392 − 0766, C530-1227 and K306-0164 are potent P-gp inhibitors that could counteract efflux and improve absorption; however, this also means these compounds must be used with care on patients undergoing multi-drug therapy, as it could lead to greater accumulation of drugs in the intracellular space^54,55^. Compounds E859-0698 and E565-0117 are neither P-gp substrates nor inhibitors, indicating a lower risk of efflux-mediated absorption problems and possibly making them more favorable for oral bioavailability (Fig. 1).

In terms of distribution, plasma protein binding (PPB) has a significant impact on the distribution and availability of a drug at the target site. All compounds have high PPB values above 96%, with C736-0680 and 6392 − 0766 showing the highest values of 99.20% and 99.00%, respectively. This high PPB indicates a lower fraction of unbound drugs available for therapeutic action, which could limit efficacy^56^. The volume of distribution (VDss) reflects the extent of distribution of the active substance in the body. Compounds 8012 − 1209 and 4896 − 4038 have positive VDss values of 0.55 and 0.529 L/kg, respectively, indicating extensive tissue distribution, which could improve efficacy. In contrast, compounds 4903 − 2142, 6392 − 0766, D361-0772, C530-1227, E565-0117, and K306-0164 have negative VDss values, suggesting limited tissue distribution and higher plasma concentrations (Fig. 1).

Excretion profiles, including clearance and half-life, are critical parameters that influence dosing frequency and risk of accumulation. Compounds C736-0680 and 8012 − 1209 have higher plasma clearance rates (CLplasma) of 4.886 ml/min/kg and 4.642 ml/min/kg, respectively, which may result in more rapid excretion and may require more frequent dosing. In contrast, E565-0117 has a lower clearance rate of 1.328 mL/min/kg, suggesting a longer retention time in the body (PMID: *32491690*). In terms of half-life (T₁/₂), D361-0772 and 8012 − 1209 have a relatively long half-life of 1.031 h and 0.795 h, respectively, suggesting a convenient dosing regimen without significant accumulation risk (Fig. 1). X77 has a shorter half-life of 0.485 h, which may require more frequent dosing to maintain therapeutic levels.

The evaluation of toxicity properties is a key step for assessing the safety profile of potential therapeutics. A major concern is cardiotoxicity, which is often associated with inhibition of the human Ether-à-go-go-Related Gene (hERG) potassium channel. Compound 4896 − 4038 has a low probability of hERG inhibition at 0.13, indicating a lower risk of cardiotoxicity^57^. In contrast, compounds 4903 − 2142, E859-0698, and K306-0164 have a higher probability of hERG inhibition with values of 0.812, 0.823, and 0.919, respectively, indicating a significant risk of cardiotoxic effects. The control drug X77 has a moderate hERG blocking probability of 0.308. In other cardiotoxicity parameters, compounds X77, E565-0117, and K306-0164 showed a probability of 0 for being related to myocardial infarction, cardiac failure, or causing arrhythmia, with compound 4896 − 4038 also being negative for the latter. These results are particularly important considering that drugs may be withdrawn from the market due to their cardiotoxic properties^58^.

The evaluation of mutagenicity and carcinogenicity is crucial for assessing long-term safety. While compound 4896 − 4038 has a high probability of genotoxicity (1), this is a common finding among the compounds studied, including X77 (0.624). However, compound 4896 − 4038 has a carcinogenicity probability of 0.773, which is lower than that of K306-0164 (0.867) but higher than that of X77. These results indicate a moderate risk of carcinogenic effects that require additional evaluation. Compounds 4903 − 2142 and E859-0698 also have high probabilities of genotoxicity and carcinogenicity, raising concerns about possible mutagenic and carcinogenic effects that require further investigation for all compounds. This overview is especially important due to the low probability of drugs being marked as carcinogenic during initial clinical trials^59^.

Other toxicity parameters were also considered. Regarding drug-induced liver injury (DILI), all compounds exhibited a high probability of causing liver damage by constant exposure, emphasizing the need for cautious interpretation and further studies to assess hepatotoxicity risks^60^. The maximum tolerated dose (MTD) for 4896 − 4038 is acceptable at 0.637 log mg/kg/day, indicating a reasonable safety margin. In addition, it has a lower risk of ototoxicity (0.634) compared to other compounds such as D361-0772 (0.865).

### Ligand geometry optimization

During our first optimization, before any DFT functionals were applied, a series of information for each compound related to their initial minimizations can be seen in Table 1.

For 4896 − 4038, 5607 pharmacophore fingerprints were identified. The ligand exhibited 125 conformations, with an average radius of gyration of 4.82 Å. The average solvent-accessible surface area (SASA) was 691.83 Å². The pair-wise RMSDs values ranged from 0.52 to 3.81 Å, with a mean value of 2.23 Å.

For 4903 − 2142, 17,457 pharmacophore fingerprints were identified. The ligand exhibited 191 conformations, with an average radius of gyration of 5.74 Å. The average SASA was 827.97 Å². The pair-wise RMSDs values ranged from 0.20 to 6.91 Å, with a mean value of 3.11 Å.

For 6392 − 0766, 4777 pharmacophore fingerprints were identified. The ligand exhibited 58 conformations, with an average radius of gyration of 5.05 Å. The average SASA was 719.04 Å². The pair-wise RMSDs values ranged from 0.33 to 3.05 Å, with a mean value of 1.85 Å.

For 8012 − 1209, 7574 pharmacophore fingerprints were identified. The ligand exhibited 134 conformations, with an average radius of gyration of 4.99 Å. The average SASA was 709.58 Å². The pair-wise RMSDs values ranged from 0.13 to 5.77 Å, with a mean value of 2.94 Å.

For C530-1227, 1,771 pharmacophore fingerprints were identified. The ligand exhibited 111 conformations, with an average radius of gyration of 4.60 Å. The average SASA was 688.69 Å². The pair-wise RMSDs values ranged from 0.10 to 3.96 Å, with a mean value of 2.07 Å.

For C736-0680, 2991 pharmacophore fingerprints were identified. The ligand exhibited 98 conformations, with an average radius of gyration of 4.83 Å. The average SASA was 656.78 Å². The pair-wise RMSDs values ranged from 0.25 to 3.57 Å, with a mean value of 1.96 Å.

For D361-0772, 9159 pharmacophore fingerprints were identified. The ligand exhibited 113 conformations, with an average radius of gyration of 5.51 Å. The average SASA was 788.03 Å². The pair-wise RMSDs values ranged from 0.08 to 5.00 Å, with a mean value of 2.32 Å.

For E565-0117, 528 pharmacophore fingerprints were identified. The ligand exhibited 1 conformation, with an average radius of gyration of 4.34 Å. The average SASA was 644.36 Å². The pair-wise RMSDs values were 0 Å, as only one conformation was present.

For E859-0698, 7708 pharmacophore fingerprints were identified. The ligand exhibited 225 conformations, with an average radius of gyration of 5.21 Å. The average SASA was 752.79 Å². The pair-wise RMSDs values ranged from 0.07 to 5.31 Å, with a mean value of 2.43 Å.

For K306-0164, 8989 pharmacophore fingerprints were identified. The ligand exhibited 93 conformations, with an average radius of gyration of 4.89 Å. The average SASA was 698.62 Å². The pair-wise RMSDs values ranged from 0.11 to 5.24 Å, with a mean value of 2.35 Å.

For X77, 6,023 pharmacophore fingerprints were identified. The ligand exhibited 106 conformations, with an average radius of gyration of 4.81 Å. The average SASA was 740.22 Å². The pair-wise RMSDs values ranged from 0.58 to 6.11 Å, with a mean value of 3.25 Å.

**Table 1:** Optimized ligand parameters for each compound.

Compound	Pharmacophores	*N*. of conf.	Avg. rad. of gyration (Å)	SASA (Å²)	Avg. RMSD (Å)
**4896 − 4038**	5607	125	4.82	691.83	2.23 (0.52–3.81)
**4903 − 2142**	17,457	191	5.74	827.97	3.11 (0.20–6.91)
**6392 − 0766**	4777	58	5.05	719.04	1.85 (0.33–3.05)
**8012 − 1209**	7574	134	4.99	709.58	2.94 (0.13–577)
**C530-1227**	1771	111	4.60	688.69	2.07 (0.10–3.96)
**C736-0680**	2991	98	4.83	656.78	1.96 (0.25–3.57)
**D361-0772**	9159	113	5.51	788.03	2.32 (0.08–5.00)
**E565-0117**	528	1	4.34	644.36	0
**E859-0698**	7708	225	5.21	752.79	2.43 (0.07–5.31)
**K306-0164**	8989	93	4.89	698.62	2.35 (0.11–5.24)
**X77**	6023	4.81	740.22	740.22	2.35 (0.58–5.24)

**Fig. 2:**
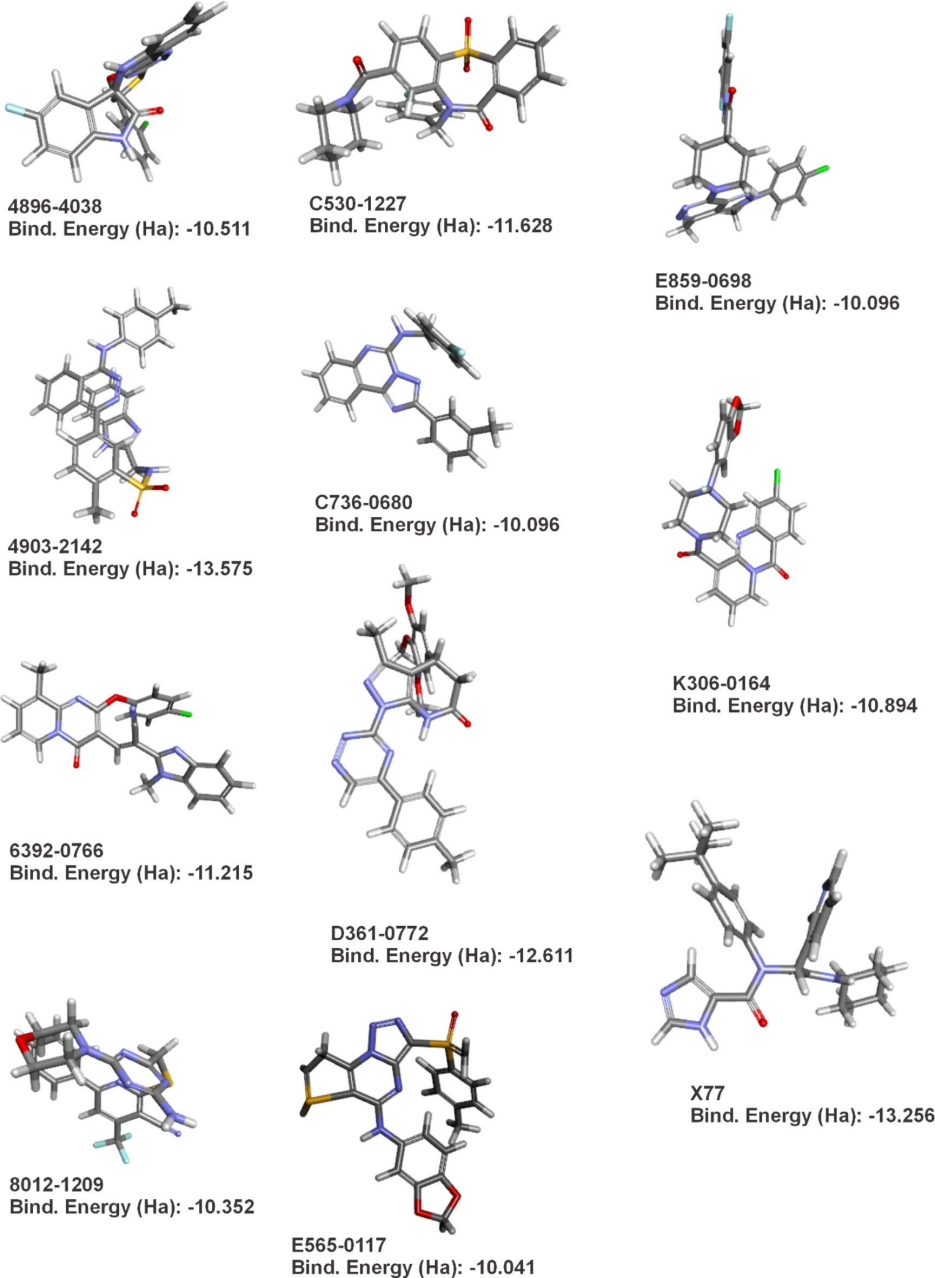
- Results of each ligand’s geometry optimization and their respective binding energy as seen in Table 2.

Afterwards, various energy-related parameters were carefully calculated for the series of eleven antiviral compounds investigated (Table 2). The total energy values for the compounds ranged from − 2291.060 Hartrees (Ha) to −1250.050 Ha, reflecting the energy of the system in its current electronic state. The binding energies, which indicate the strength of the interaction between each compound and the SARS-CoV-2 main protease (Mpro), varied between − 10.041 kcal.mol^−1^ and − 13.575 kcal.mol^−1^. The result of each optimization and the respective binding energies for each compound can be seen in Fig. 2.

The dipole moments of the compounds, which indicate charge separation within the molecules and may influence solubility and intermolecular interactions, were measured to be between 1.408 Debye and 8.905 Debye (Table 2). The dielectric energy, which reflects the polarization energy in a dielectric medium, and the solvation energy, which indicates the change in energy as the molecule passes from a vacuum to a solvent, were calculated for each compound, with values ranging from − 0.028 Ha to −0.121 Ha (Table 2).

The surface areas of the molecules, which provide information on the extent of their interaction with the biological environment and the target enzyme, ranged from 1332.771 Å² to 1676.061 Å². In addition, cavity volumes between 2770.448 cubic angstroms and 3669.172 cubic angstroms were determined, measuring the space within the molecular structures and their potential to interact with the active sites of enzymes (Table 2).

**Table 2:** Quantum energies table for all 10 anti-mpro compounds and reference drug X77.

Quantum Energies (Ha)										
Compound	Total Energy	Binding Energy	HOMO Energy	LUMO Energy	Band Gap Energy	Dipole Mag	Dielectric Energy	Solvation Energy	Surface Area	Cavity Volume
4896 − 4038	−2291.060	−10.511	−0.208	−0.126	0.082	5.553	−0.054	−0.054	1449.619	3196.112
4903 − 2142	−2020.530	−13.575	−0.190	−0.106	0.084	7.602	−0.083	−0.083	1577.670	3669.172
6392 − 0766	−1872.280	−11.215	−0.209	−0.131	0.078	4.838	−0.052	−0.052	1508.533	3262.979
E859-0698	−2015.570	−10.096	−0.198	−0.087	0.110	1.408	−0.028	−0.028	1332.771	2770.448
C736-0680	−1250.050	−10.096	−0.198	−0.087	0.110	1.408	−0.028	−0.028	1332.771	2770.448
E565-0117	−2131.050	−10.041	−0.184	−0.112	0.072	8.905	−0.082	−0.082	1468.311	3202.080
8012 − 1209	−1953.240	−10.352	−0.207	−0.137	0.070	3.974	−0.042	−0.042	1466.383	3152.826
K306-0164	−1892.900	−10.894	−0.215	−0.128	0.087	5.612	−0.121	−0.121	1445.999	3144.726
D361-0772	−1621.870	−12.611	−0.181	−0.131	0.050	3.335	−0.062	−0.062	1676.061	3504.438
C530-1227	−1898.920	−11.628	−0.227	−0.114	0.113	4.268	−0.055	−0.055	1458.770	3260.545
X77	−1460.140	−13.256	−0.213	−0.077	0.136	2.974	−0.049	−0.049	1593.295	3451.215

### Molecular Docking

**Fig. 3:**
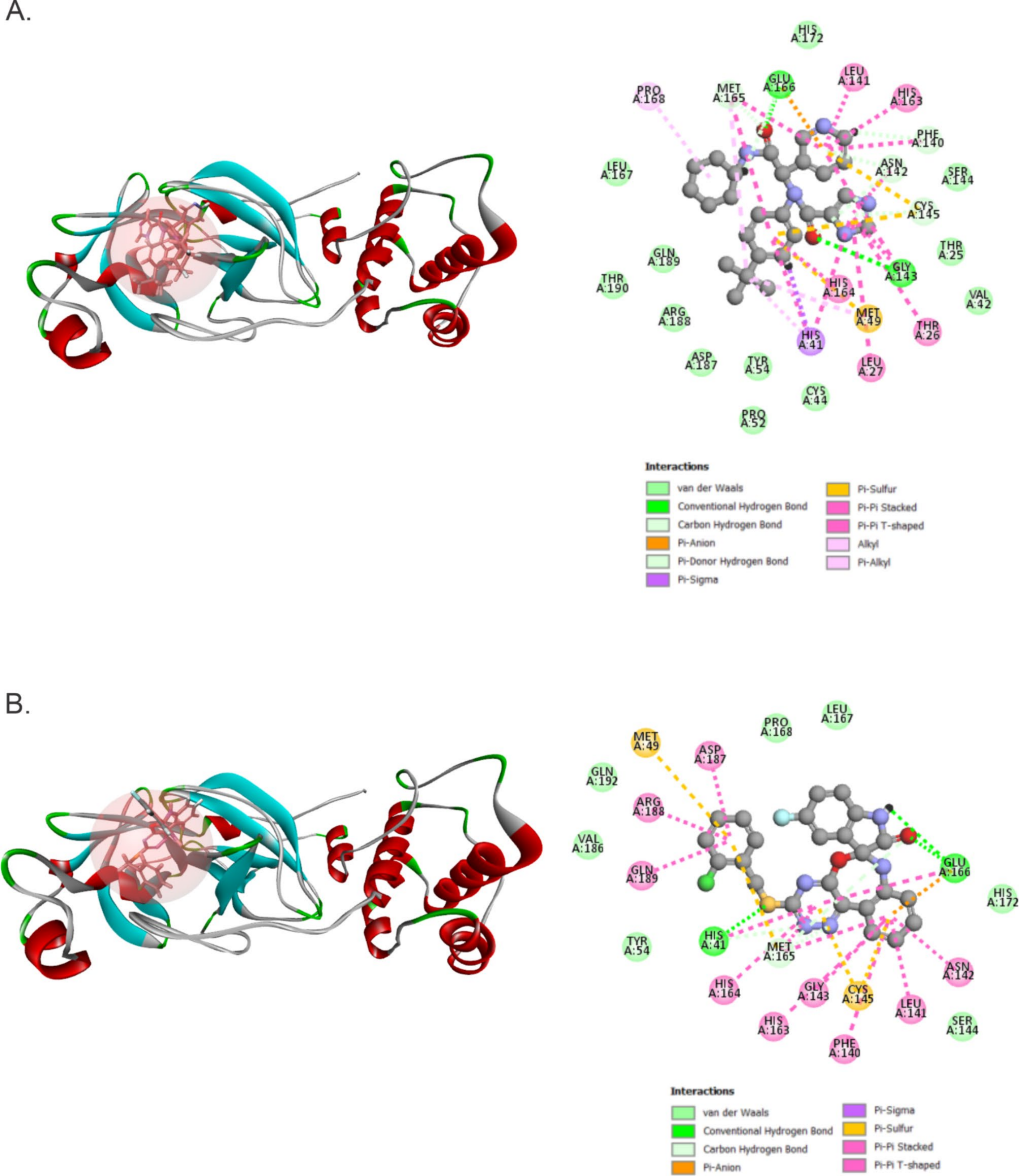
- Protein-ligand complexes after molecular docking. **(A)** Docked pose of ligand X77 in Mpro and its respective intermolecular binding forces. **(B)** Docked pose of ligand 4896 − 4038 in Mpro and its respective intermolecular binding forces.

The current computational study focuses on identifying new potential inhibitors for Mpro from SARS-CoV-2 and analyzing their binding patterns using structure-based screening and molecular dynamics simulation studies to determine their structural properties. Designing compounds by using the structures of target proteins and the active binding molecules of protein residues is a crucial initial step in developing geared therapeutics. We have successfully screened 2976 components with reference compound X77. Table 2 and Fig. 2 show the optimized spatial geometry of the 10 best ligands and highlight the internal binding energy as well as the electronic and structural parameters. The main protease (Mpro/3CLpro) is a vital enzyme in the replication of coronaviruses, with a crucial proteolytic function cleaving viral polyprotein into functional proteins^61^. The protein of interest, identified by its PDB ID 6W63, has been chosen as the receptor for conducting docking studies (Table 3). The validation process involved redocking the native ligand (X77) onto the target protein. The active substances mostly form stable interactions with the receptor through hydrogen bonding and hydrophobic interactions. The ligands that attach to the active site of this receptor can greatly impede the functioning of the protein. From this point on, for our binding properties analysis and molecular dynamics studies, we will focus our attention on two ligands: 4896 − 4038, which displayed the best ADMET profiles and thus is a good candidate when taking into account major health concerns on patients, and X77, our control drug. According to our docking analysis, X77 exhibited a interaction energy (Swiss Param Score) of −53.42 (–9.42) kcal.mol^−1^, with its main energy contributions coming by forming of hydrogen bonds with Glu166 and Gly143, as well as carbon hydrogen bonds with Met165, Asn142, Cys145 and Phe140 (Fig. 3A). These hydrogen bonds have been reported in other works employing similar molecular docking and dynamics methods, and thus are in accordance with current literature in explaining the high binding energies found in the Mpro-X77 complex^62,63^. In fact, Stille et al. confirmed the existence of these bonds to Gly143 through molecular docking and molecular dynamics, as well as their contribution to the overall binding energies in the Mpro-X77 complex, whereas Jiang et al. further explained the binding contributions of the hydrogen bonds between X77 and Mpro residues Gly143 and Glu166, thus corroborating with our findings and reinforcing the validity of our in silico findings through reproducibility^62,63^.

On the other hand, 4896 − 4038 presented a interaction energy (Swiss Param Score) of 40.30 (−9.22) kcal.mol^−1^, and strong bonds with Glu166 and His41, as well as carbon hydrogen bonds with Met165 (Fig. 3A). Both ligands presented multiple Pi-Pi and Pi-Sigma bonds, with a singular Pi-Sulfur bond being formed between X77 (4896 − 4038) and Met49 (Met49 and Cys145). (Figure 3A and B).

**Table 3:** Best docking scores and parameters during score ligand poses analysis.

Molecular Docking Analysis											
Features	4896 − 4038	4903 − 2142	6392 − 0766	8012 − 1209	C530-1227	C736-0680	D361-0772	E565-0117	E859-0698	K306-0164	X77
AC Score	−7,80	46,70	15,47	27,16	−89,81	49,82	109,20	77,31	63,12	10,09	−9,98
SwissParam Score	−8,25	−9,22	−8,38	−8,90	−8,82	−8,64	−8,86	−8,83	−9,21	−8,97	−9,42
Affinity	−8,53	−10,07	−9,97	−8,12	−9,26	−9,43	−9,42	−9,13	−9,39	−7,87	−7,08
Total Energy	65,49	69,93	31,49	22,37	53,09	50,79	90,56	38,48	62,28	60,31	92,46
Internal Energy	−30,53	−42,29	−36,74	−19,98	−37,60	−31,22	−35,33	−36,35	−34,30	−35,78	−28,43
vdW Energy	−27,53	−38,47	−35,91	−18,38	−29,07	−30,34	−30,79	−33,70	−32,79	−16,81	−15,06
Electrostatic Energy	−3,00	−3,82	−0,83	−1,61	−8,53	−0,88	−4,54	−2,65	−1,51	−18,97	−13,38
﻿Solvent Accessibility	571,45	759,83	690,86	553,43	622,25	579,57	674,90	615,23	684,92	574,51	688,39
Interaction Energy	−40,30	−49,80	−40,05	−46,30	−44,20	−42,39	−46,97	−43,80	−47,40	−47,38	−53,42

### Molecular Dynamics Simulation analysis

A MD simulation was conducted for the antiviral compound 4896 − 4038 and the control drug X77 both had a duration of 300 ns. Various factors can impact in silico predictions in an erratic manner, including the shape of the ligand, the presence of water molecules, ions, co-factors, the protonation state of the ligand, and the entropies related to conformational changes and solvation^64^. Multiple reports were produced to authenticate the docking outcomes^65,66^. MD was used to ascertain the stability of the interactions within ligand-protein docked complexes and to compare with the co-crystallized complex.

**Fig. 4A:**
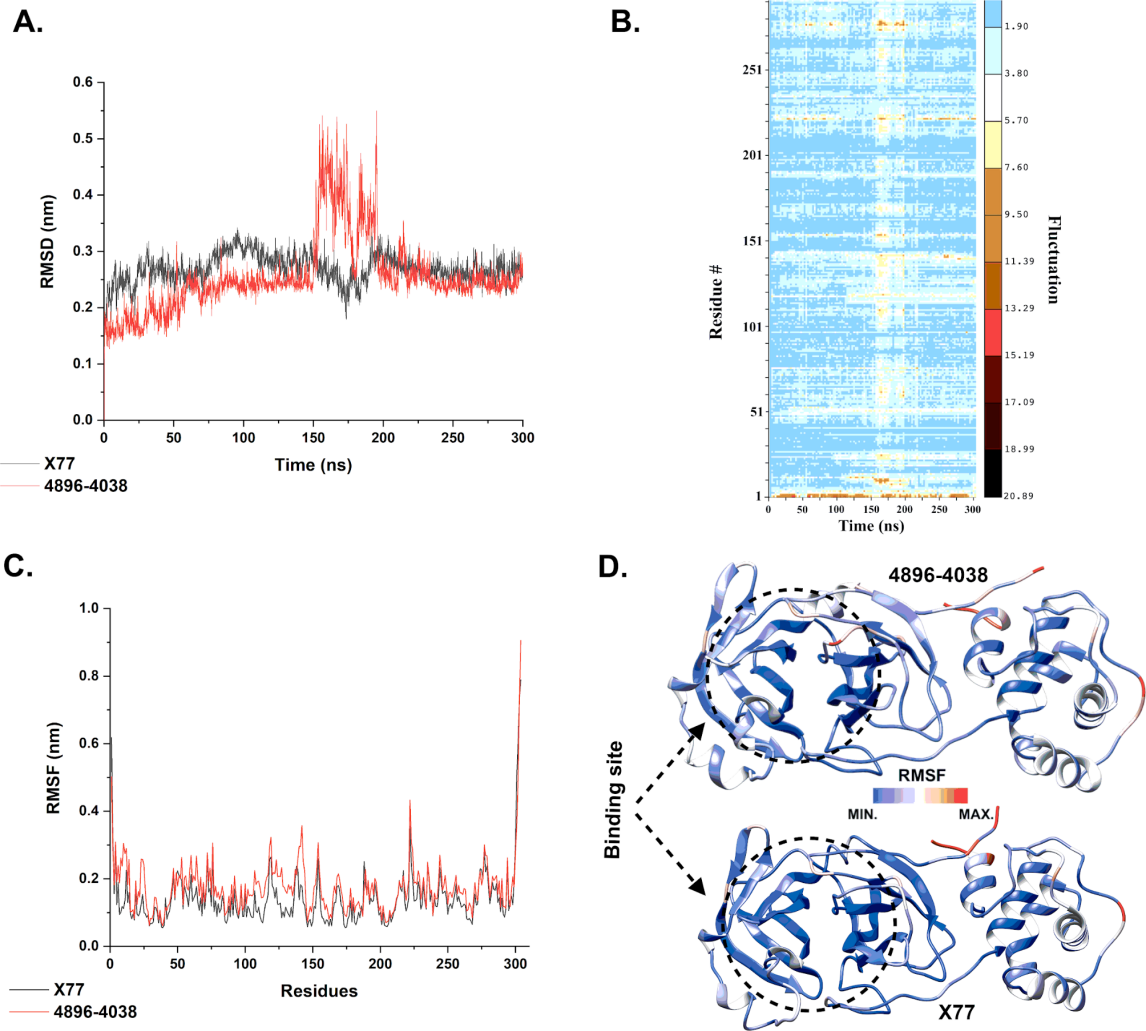
displays the time-dependent RMSD values of the C-alpha atoms in relation to the complex structure, which were computed during MD simulations. It can be observed that in the first 150 ns of the simulation, the Mpro/X77 complex is more stable than the Mpro/4869 − 4038 complex. The Mpro/4869 − 4038 complex shows an increased RMSD value (up to 0.5 nm) between 150–200 ns, while the Mpro/X77 complex shows a slight decrease in the RMSD value (from 0.3 to 0.2 nm). However, both complexes stabilize similarly around 0.25 nm after 200 ns simulations, which is a very low value for RMSD analysis. These results indicate that the simulation time was able to obtain stable protein-ligand complexes.

In Fig. 4**(A)**, the root mean square deviations (RMSD) are plotted as a function of molecular dynamic (MD) simulation time (300 ns) for ligands X77 and 4869 − 4038. The RMSD of the protein for the c-alpha atoms is represented by the black and red lines for X77 and 4896 − 4038 compounds, respectively. **(B)** Heatmap of the residue fluctuation as a function of time for the complex Mpro/4869 − 4038. **(C)** The root mean square fluctuation (RMSF) per residue considering the whole protein for the calculation. The RMSF of the protein is represented by the black and red lines for compounds X77 and 4896 − 4038. **(D)** Three-dimensional representation of the Mpro protein colored by the RMSF values for both complexes. Red color means high fluctuation and blue color means low fluctuation.

To understand the main reason for the increased RMSD of the Mpro/4869 − 4038 complex between 150 and 200 ns, we analyzed the fluctuation per residue as a function of time (Fig. 4B). It can be seen that the fluctuation does indeed increase throughout the protein during this period (yellow shades), especially near the N- and C-terminals. However, it is not a significant fluctuation that causes a shift in the protein conformation. In fact, both analyzes (Fig. 4A and B) show that the protein has returned to a more stable conformation.

Root Mean Square Fluctuation (RMSF) analysis (Fig. 4C) of the two complexes shows the similarity between them. The RMSF profile is very similar, with most values below 0.3 nm, indicating very stable proteins. The RMSF analysis indicates that while the N- and C-termini are highly flexible, each ligand’s binding site remains largely stable (see Fig. 4D). It is plausible that the flexibility of the termini makes the motions more flexible, which may aid in ligand binding and show a dynamic interaction between the ligand and protein. Such adaptability may improve the efficiency of ligand binding and location, which is crucial for the development of potent inhibitors^67–70^.

**Fig. 5:**
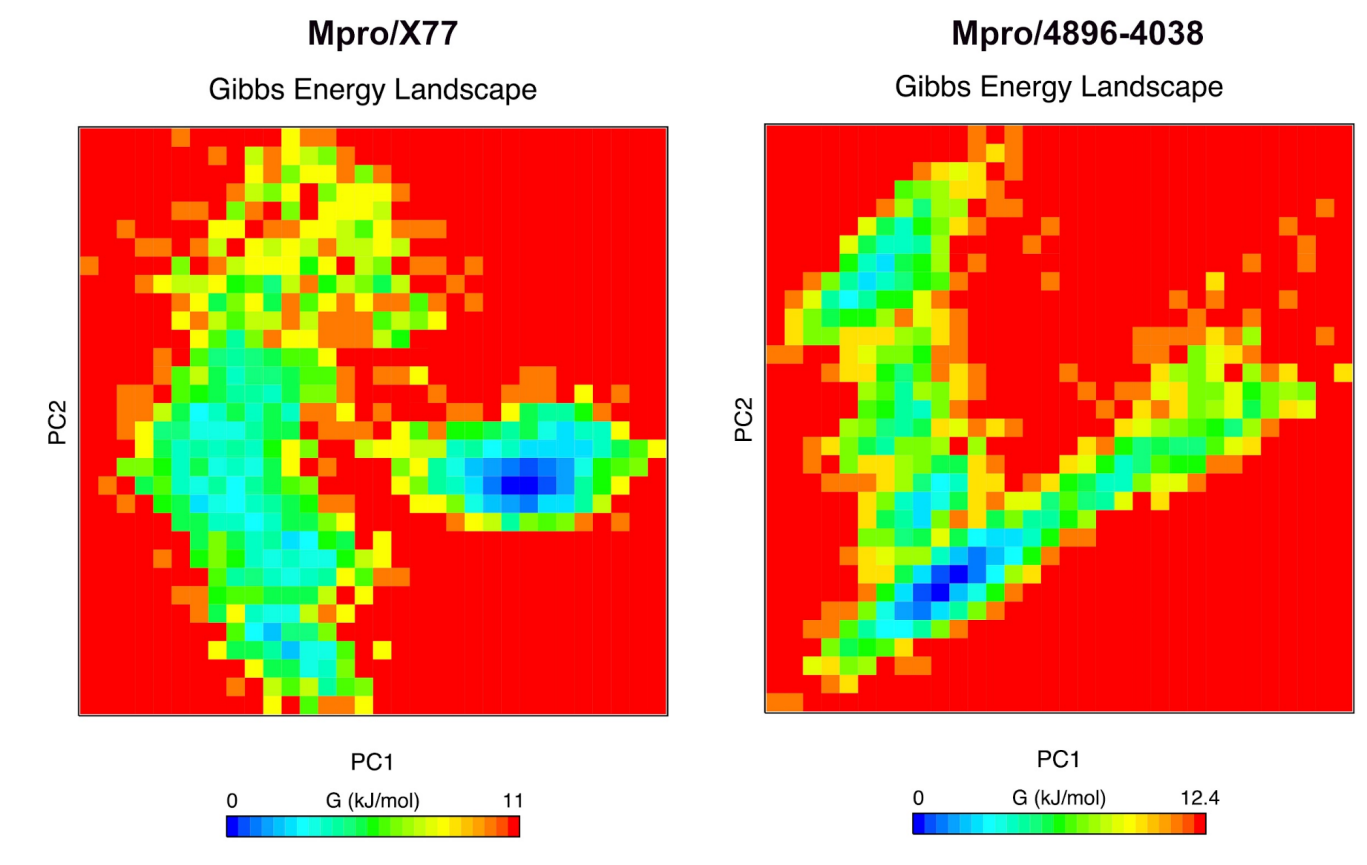
**-** Free energy landscape (FEL) and principal component analysis (PCA) of the protein-ligand complex. The figure shows the FEL obtained from the molecular dynamics (MD) simulation of the protein-ligand complex. The PCA was used to reduce the dimensionality of the data, with principal components PC1 and PC2 representing the directions of the largest conformational variations. The blue areas represent stable conformations with lower free energy, while the red areas show less stable conformations with higher energy.

Principal component analysis (PCA) was performed to reduce the dimensionality of the data obtained from molecular dynamics (MD) simulation and to identify the main modes of motion of the protein-ligand complex. This technique allows simplifying the analysis and visualizing the main conformational transitions during the simulation. For this purpose, we have considered the entire protein and ligand structures. The principal components (PC1 and PC2) were calculated (Fig. 5), which represent the directions of the largest variation in the structure of both complexes. PC1 captures most of the variation in the structural movements, while PC2 complements this information by representing the second largest source of variation. We have also added the Free Energy Landscape (FEL) analysis created by mapping the Gibbs free energy as a function of the PC1 and PC2 principal components. This analysis gives a detailed insight into the stability of the complex conformations during the simulation.

As can be seen in Fig. 5, the Mpro/X77 complex has two different clusters, one of which has a minimum in dark blue. The other cluster has a minimum with a low light blue region and predominantly green regions. The Mpro/4869 − 4038 complex, on the other hand, shows only a very pronounced minimum (dark blue region), which suggests that it is more stable than the Mpro/X77 complex. However, the difference between the two complexes does not appear to be very large.

In addition, we performed the MM/PBSA calculation with a decomposition analysis to see the complex energy along the last 50 ns of the simulation and the residues that contributed the most to the binding energy with the ligands. The calculated terms for MM/PBSA can be seen in Table 4. It can be seen that the average total energy (ΔE_Total_) for both complexes was negative, indicating that these complexes are energetically favorable. The difference in total energy (ΔE_Total_) was small, with X77 at − 4.91 kcal.mol^–1^ and − 6.89 kcal.mol^–1^ for 4869 − 4038. In contrast to ΔE_vdw_, a significant difference in term energies was observed between both complexes. These calculations indicate a slightly higher stability of Mpro/4869 − 4038 compared to Mpro/X77.

**Table 4:** Binding free energy (Kcal.mol^−1^) for the selected compound of Mpro and its related co-crystal X77.

Compound	ΔE_vdw_	ΔE_ele_	ΔE_PB_	ΔE_*N*−polar_	ΔE_Disper_	ΔG_gas_	ΔG_solv_	ΔE_Total_
X77	−48.82	−8.02	27.02	−32.65	57.56	−56.84	51.93	−4.91
4869 − 4038	−48.70	−0.36	18.21	−25.25	49.20	−49.06	42.16	−6.89

**Fig. 6:**
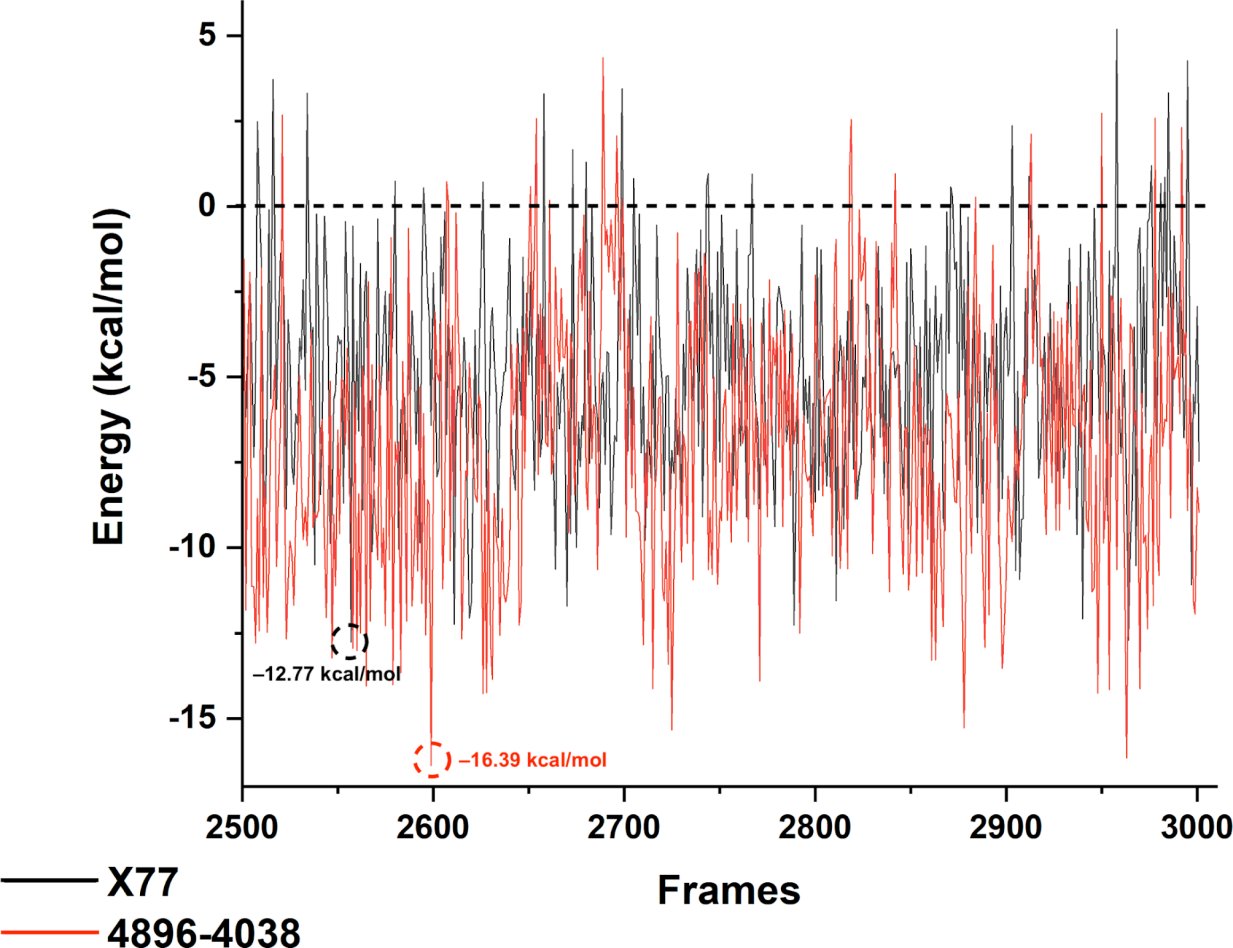
**-** Free binding energy from the MM/PBSA calculation from the last 500 frames (50 ns) of each simulation. The black line represents the energy of the Mpro/X77 complex and the red line corresponds to Mpro/4896 − 4038.

The MM/PBSA values per frame can be seen in Fig. 6. It can be seen that most of the energies for both complexes were negative values and only a few frames had an energy above 0. The lowest value for Mpro/X77 was − 12.77 kcal.mol^–1^ corresponding to frame 2557, while the highest value was 5.19 kcal.mol^–1^ from frame 2958. For the complex Mpro/4869 − 4038, the lowest and highest energies were − 16.38 kcal.mol^–1^ and 4.36 from frames 2599 and 2689, respectively. In general, it can also be seen that the red line (Mpro/4869 − 4038) has lower energy values, which is consistent with the previous analysis indicating a more stable complex compared to Mpro/X77.

**Fig. 7:**
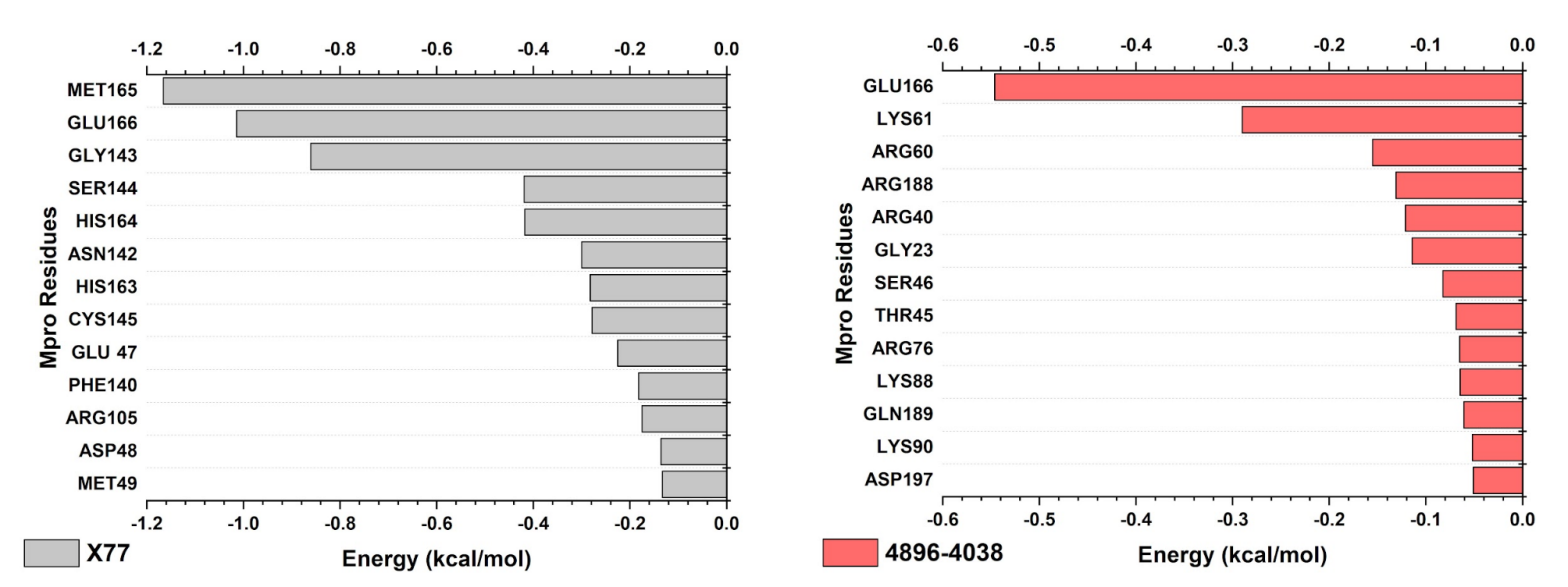
**-** Per residue decomposition energy analysis showing the amino acids that contribute most to binding affinity with ligands X77 and 4896 − 4038.

The decomposition analysis, in which the energy contribution per residue is evaluated, shows the different pattern of residues contributing most to the binding energy depending on the ligand (Fig. 7). For the Mpro/X77 complex, the descending order of the binding affinity of the residues and the respective energy in kcal.mol^–1^ was: Met165 (−1.166) > Glu166 (−1.015) > Gly143 (−0.861) > Ser144 (−0.419) > His164 (−0.418) > Asn142 (−0.300) > His163 (−0.283) > Cys145 (−0.278) > Glu47 (−0.225) > Phe140 (−0.182) > Arg105 (−0.175) > Asp48 (−0.136) > Met49 (−0.133). For Mpro/4896 − 4038, these residues were: Glu166 (−0.546) > Lys61 (−0.290) > Arg60 (−0.155) > Arg188 (−0.131) > Arg40 (−0.121) > Gly23 (−0.114) > Ser46 (−0.082) > Thr45 (−0.069) > Arg76 (−0.065) > Lys88 (−0.065) > Gln189 (−0.061) > Lys90 (−0.052) > Asp197 (−0.051). The only common residue among these was Glu166, which contributed the most to the affinity energy of Mpro/4896 − 4038 and the second most in the Mpro/X77 complex. This could explain the difference in stability between these complexes.

## Discussion

The analysis of the physicochemical and ADMET properties of the antiviral compounds provided important insights into their potential as SARS-CoV-2 Mpro inhibitors, and are a crucial step in analyzing the pharmacokinetic profile of new drugs^71^. The evaluations focused on key parameters such as MW, LogP, hydrogen bonding capacity, tPSA, and compliance with Lipinski’s Rule of Five and Veber’s Rule, which are critical for assessing drug-likeness and oral bioavailability^48,50,72^. Most compounds complied with these rules, indicating favorable profiles for oral administration. In particular, compounds C530-1227 and 8012 − 1209 exhibited promising drug-like properties with high QED values and acceptable pharmacokinetic criteria, suggesting that they are suitable for further investigation.

In terms of absorption, the compounds showed varying degrees of permeability and absorption in the human intestinal absorption. Compounds such as D361-0772 and K306-0164 showed excellent absorption values in the human intestinal absorption evaluation, indicating a high potential for oral bioavailability. However, for several compounds, including 4896 − 4038 and C736-0680, interactions with P-glycoprotein (P-gp) were observed, which could affect absorption due to efflux mechanisms and impact overall efficacy, although the inhibitory nature of 4896 − 4038 could increase its effects^54,55^.

Regarding distribution, all compounds exhibited high plasma protein binding values, suggesting a lower proportion of unbound drug available for therapeutic action^56^. Nevertheless, compounds such as 4896 − 4038 and 8012 − 1209 showed positive values for volume of distribution, indicating extensive tissue distribution that could increase their therapeutic efficacy. This extensive distribution is beneficial for reaching the target sites in the body.

The excretion profiles illustrated the differences in the excretion rates and half-lives of the individual substances. Compounds such as E565-0117 had lower plasma clearance rates, which could lead to longer retention times and less frequent dosing requirements. In contrast, compounds with higher clearance rates, such as C736-0680 and 8012 − 1209, might require more frequent dosing to maintain therapeutic levels (PMID: 32491690).

Toxicity assessments revealed potential concerns, particularly with regard to cardiotoxicity and genotoxicity. Compound 4896 − 4038 was characterized by a lower likelihood of hERG inhibition, indicating a lower risk of cardiotoxic effects compared to other candidates^57^. Although the compound had a high likelihood of genotoxicity, this was a common feature of the compounds studied, including the reference drug X77. The maximum tolerated dose for 4896 − 4038 was acceptable, suggesting a reasonable safety margin for further development.

In summary, 4896 − 4038 was the most promising candidate due to its balanced profile of physicochemical and pharmacokinetic properties and its relatively favorable safety profile. Compared to the reference drug X77, 4896 − 4038 offers potential advantages, including higher binding affinity and stability. These results suggest that 4896 − 4038 is a strong candidate for further development as a SARS-CoV-2 Mpro inhibitor with the potential to make an important contribution to therapeutic strategies against COVID-19.

In the geometric analysis, compounds X77, 4903 – 2142, and E859 – 0698 exhibited the most favorable internal binding energies. Interestingly, in subsequent docking studies, these same compounds also showed high docking scores. Such findings suggest that the geometric stability of the ligands—ensured by quantum-level protocols for minimizing internal energies and intramolecular interactions—may contribute to more efficient docking at the target receptor. Nevertheless, this conclusion remains hypothetical and requires further investigation for confirmation.

Another key factor influencing molecular stability and reactivity is the HOMO-LUMO gap energy. A smaller gap generally indicates a more reactive compound, while a larger gap suggests stability, which is advantageous for pharmacological applications^73^. Compound X77 exhibited the largest gap energy (0.136), indicating favorable stability that may enhance its pharmacokinetic profile. Similarly, 4903 − 2142 also had a relatively high gap energy (0.084), balancing reactivity with a level of stability suitable for drug-like behavior.

Our molecular docking evaluated a multitude of compounds in regard to their interaction with the SARS-CoV-2 Mpro. During our docking analysis, the interaction energy profiles of each compound provided key insights into their interaction with the protein, as more favorable interaction energy values generally indicating stronger interactions. Among the compounds analyzed, X77 exhibited the highest interaction energy (53.42), indicating a strong and potentially stable binding interaction with the target site. This result agrees well with other favorable results of the docking analysis and underlines the potential of X77 as a high-affinity ligand, and are on par with other literature findings for this compound^62,63^.

Other compounds such as 4903 – 2142 (IE = − 49.80 kcal.mol^−1^; SPS = − 9.22 kcal.mol^−1^), E859 – 0698 (IE = − 47.40 kcal.mol^−1^; SPS = − 9.21 kcal.mol^−1^) and K306–0164 (IE = − 47.38 kcal.mol^−1^; SPS = − 8.97 kcal.mol^−1^) also exhibited relatively high interaction energies, suggesting that these compounds may have similarly stable interactions within the binding pocket. Although their interaction energies are slightly higher than those of X77, they still fall within a range suggestive of robust binding potential. This could render these compounds promising candidates for further investigation, as their binding properties may facilitate effective inhibition or modulation of the target protein. Nonetheless, it must be reiterated that none of them exhibit the favorable ADMET parameters observed for compound 4896–40–38, as indicated by our simulations.

The MD analysis shows that the Mpro/X77 complex is more stable than the Mpro/4869 − 4038 complex during the first 150 ns of the MD simulation, with the latter experiencing increased RMSD values between 150 and 200 ns. Both complexes stabilize around 0.25 nm after 200 ns, indicating stable protein-ligand interactions. The RMSF analysis indicated that both complexes have similar stability, with most values below 0.3 nm, except for higher fluctuations at the N- and C-terminals. The 3D structure colored by RMSF values confirms stable binding sites for both ligands, suggesting no significant difference in binding site stability. These results are very similar with previous MD simulations of Mpro protein, which suggests that the observations of these studies are reliable^74,75^.

Further results were drawn from the PCA-assisted analysis, which revealed that Mpro/X77 has greater conformational flexibility than Mpro/X77, which had two clusters during the Free Energy Landscape (FEL) analysis. These clusters show that the simulation contains a large number of Mpro/X77 conformations, which may provide the ligand with additional interactions. In contrast, the Mpro/4869 − 4038’s stiffness showed a single deep minimum on the FEL, suggesting that there is only one stable conformation with the possibility of the ligand forming stronger connections^76,77^.

These results are consistent with those of Liang et al. (2022)^78^, who demonstrated the dynamic nature of Mpro with various inhibitors through extensive MD simulations and emphasized the importance of conformational flexibility in inhibitor binding. Similarly, Kneller et al. (2020)^79^emphasized the conformational adaptability of Mpro as crucial for its function and interaction with inhibitors. The pronounced stability of the Mpro/4869 − 4038 complex, as evidenced by the single deep minimum in the FEL, suggests a strong and potentially more specific interaction compared to Mpro/X77. The study by Douangamath et al. (2020)^80^ which identified several potent inhibitors of Mpro, emphasized the need for stable binding interactions to achieve effective inhibition as observed for the 4869 − 4038 ligand.

In the study, MM/PBSA calculations with decomposition analysis were performed to evaluate the complex energy during the last 50 ns of the simulation and to identify the residues that contributed most to the binding energy with the ligands. The calculated MM/PBSA terms show that the average total energies (ΔE_Total_) were negative for both complexes, indicating that these complexes are energetically favorable. The difference in ΔE_Total_ between the complexes was small, with − 4.91 kcal.mol^–1^ for X77 and − 6.89 kcal.mol^–1^ for 4869 − 4038.

However, the most striking difference that emerged from the MM/PBSA was found when analyzing the energy decomposition per residue. The X77 ligand has a high binding affinity to Cys145, which is described as a crucial residue of the catalytic dyad for the activity of Mpro^81,82^. Although the ligand 4869 − 4038 does not show a high affinity for the amino acids of the catalytic dyad (His41 and Cys145) in this analysis, it shows a strong interaction with Glu166. Several studies have reported on the importance of Glu166 and described this residue as crucial for ligand binding and the stability of the complex^83,84^. It is important to emphasize the MM/PBSA limitation, which may not describe the most important interactions of each complex. More detailed analysis, such as quantum mechanical analysis, should be performed to confirm these important amino acid residues.

## Conclusion

This work utilized the newly available crystal structure of the SARS-CoV-2 Mpro protein to perform comprehensive searches for repurposing drugs at many scales. The data unequivocally established the interaction between 4896 − 4038 and X77. The ChemDiv chemical library was utilized to screen for human protease antiviral agents. A regression model was employed for this screening process. The compounds that showed promising results were then subjected to docking with the COVID-19 Mpro protein. The binding stability and conformational changes of the protein-ligand complexes were observed using molecular dynamics investigations conducted on the top-screened compound and the co-crystalized control X77 compound. Physicochemical and ADMET profiles analysis showed that compound 4869 − 4038 is a promising candidate for Mpro targeting. It fulfilled the most important physicochemical criteria, such as an acceptable molecular weight and log P values, indicating a balance between solubility and membrane permeability. Crucially, 4896 − 4038 had a relatively favorable safety profile. Compared to other candidates, it showed a lower likelihood of hERG inhibition, suggesting a lower risk of cardiotoxic effects, an important concern in drug development. Furthermore, quantum chemical analysis and molecular docking studies showed that although compounds such as 4903 − 2142 and X77 had very negative binding energies and strong interaction energies— - suggesting robust binding affinity to Mpro — compound 4896 − 4038 provided a superior balance between binding affinity and overall pharmacokinetic and safety profiles. Its HOMO-LUMO gap energy suggests sufficient molecular stability to balance reactivity with a favorable pharmacokinetic profile. MD analysis revealed that both complexes are stable in solution during the 300 ns of the simulation. PCA and FEL also showed a slightly more stable Mpro/4869 − 4038 complex compared to the Mpro/X77 complex. The MM/PBSA calculations were consistent with these results when the average binding affinity of Mpro/4869 − 4038 was slightly lower than that of Mpro/X77. In summary, these results indicate that both ligands are suitable for an inhibitory effect on Mpro. Therefore, these two compounds are proposed for validation and future preclinical and clinical investigation as more effective drugs for COVID-19.

## Data Availability

The datasets used and/or analyzed during the current study are available from the corresponding author on reasonable request.
